# Rapid diagnosis of *Aspergillus flavus* infection in acute very severe aplastic anemia with metagenomic next-generation sequencing: a case report and literature review

**DOI:** 10.3389/fmed.2024.1413964

**Published:** 2024-09-23

**Authors:** Ying Kang, Xiaojing Zhang, Cao Qin, Yafeng Zheng, Wei Gai, Xiaofei Jia, Bo Shao, Shuai Zhang, Hao Jiang, XiaoJun Huang, Jinsong Jia

**Affiliations:** ^1^Peking University Institute of Hematology, Peking University People's Hospital, Beijing, China; ^2^WillingMed Technology Beijing Co., Ltd., Beijing, China; ^3^Beijing Qinghe Hospital, Beijing, China

**Keywords:** *Aspergillus flavus*, invasive fungal infection, aplastic anemia, metagenomic next-generation sequencing, blood were negative

## Abstract

Infection remains the leading cause of mortality in severe aplastic anemia (SAA) patients, with invasive fungal infections being the great threat. *Aspergillus fumigatus* accounts for most of the reported fungal infection cases. Here, we present a case of *A. flavus* infection in a patient with acute very severe aplastic anemia (VSAA) despite persistently negative clinical fungal tests. The patient was admitted to the hospital due to pancytopenia presisting for over a month and intermittent fever for 10 days. Elevated inflammatory indicators and abnormal lung imaging suggested infection, prompting consideration of fungal involvement. Despite negative results from multiple blood, sputum fungal cultures and the serum (1,3)-β-D-glucan/galactomannan tests. Metagenomic next-generation sequencing (mNGS) on multiple blood samples, alongside clinical symptoms, confirmed *A. flavus* infection. Targeted antifungal treatment with liposomal amphotericin B and voriconazole significantly ameliorated pulmonary symptoms. Additionally, this study reviewed and compared the symptoms, diagnostic approaches, and treatments from prior *Aspergillus* infections in AA patients. It emphasizes critical role of early mNGS utilization in diagnosing and managing infectious diseases, offering insights for diagnosing and treating fungal infections in VSAA.

## 1 Introduction

Aplastic anemia (AA) is characterized by bone marrow failure, leading to abnormal proliferation of cells and peripheral blood pancytopenia. Neutrophil deficiency increases infection risk, the primary cause of death in severe AA (SAA), with invasive fungal infection posing the highest mortality (1, 2). Prompt restoration of neutrophil levels is crucial in SAA treatment, preceded by infection control (1, 3). Common fungal infections in AA include *Aspergillus*, *Zygomycetes*, *Candida*, and *Fusarium*, while gram-positive bacteria such as *Staphylococci* and *Enterococci*, and gram-negative bacteria *Escherichia coli*, *Klebsiella pneumoniae*, *Pseudomonas aeruginosa*, and gram-negative bacilli are prevalent pathogens. Viral infections, primarily herpesviruses and hepatitis viruses, are less common (2, 4).

Invasive aspergillosis poses a significant mortality risk in immunocompromised patients (5), with neutropenia being a key risk factor (6). Diagnosis is challenging due to variable and non-specific clinical presentations. Metagenomic next-generation sequencing (mNGS) is an emerging technique for detecting all microorganism nucleic acids in clinical samples, improving invasive pulmonary aspergillosis (IPA) diagnosis (7–9), and enhancing detection of viruses, and uncommon pathogens (10, 11). Moreover, mNGS offers rapid, precise pathogen detection, complementing clinical diagnostics (12, 13).

The purpose of this study is to provide assistance in the diagnosis and treatment of patients with invasive fungal aplastic anemia by presenting a rare case of *Aspergillus* infection and a literature review of previous studies. This study reports a case with recurrent very severe AA [VSAA, with absolute neutrophil count (ANC) < 0.2 × 10^9^/L, and bone marrow (BM) cellularity less than 25% (or less than 50% if BM is comprised of less than 30% hematopoietic cells)] (14), diagnosed with *Aspergillus flavus* infection through multiple mNGS tests despite negative cultures. Targeted treatment improved the patient’s condition, demonstrating the efficacy of mNGS in early diagnosis. We also reviewed previously cases of *Aspergillus* infection in adult AA patients to deepen understanding of this condition. To our knowledge, this represents a rare reported case.

## 2 Case description

**Figure 1:**
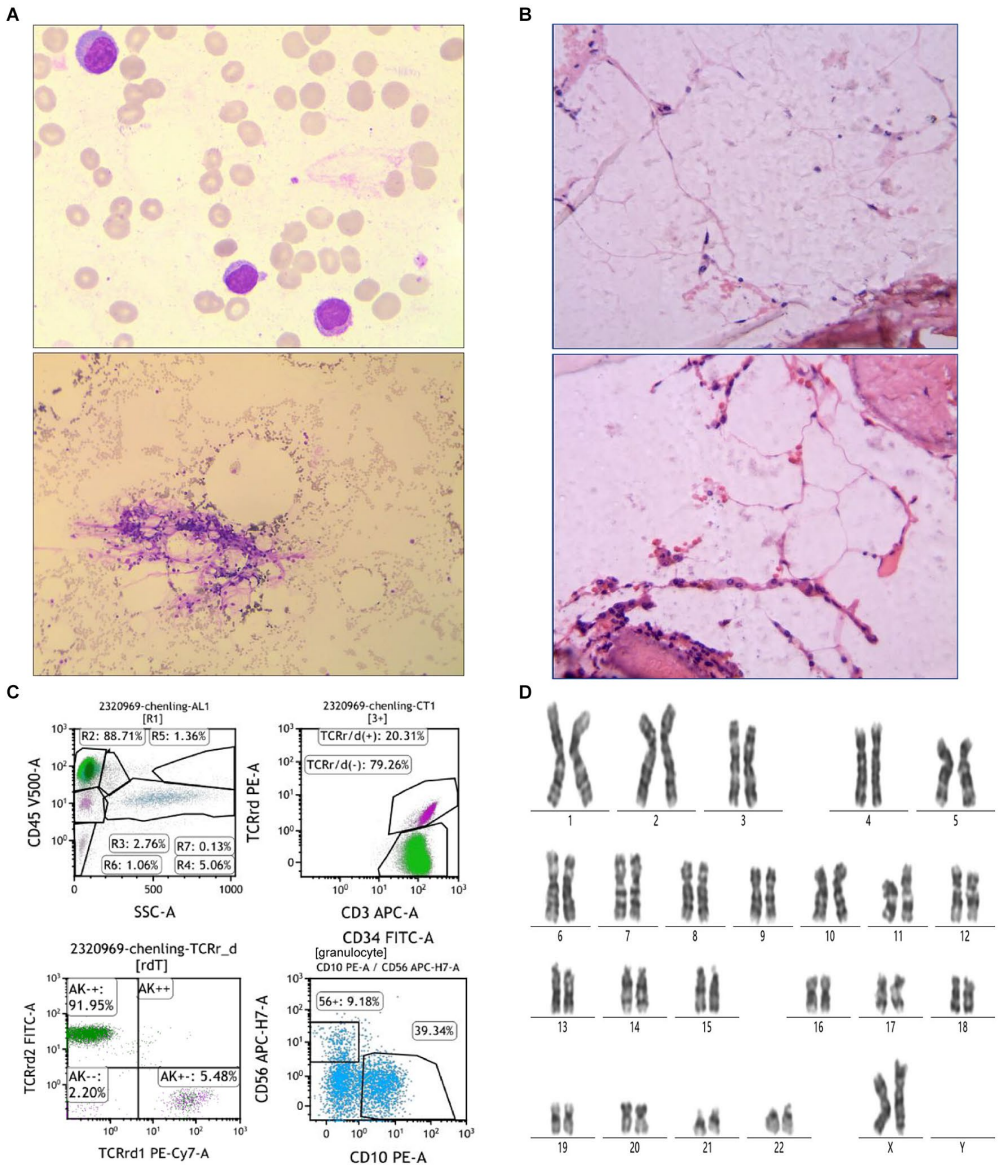
Patient bone marrow aspiration and biopsy results. **(A)** Bone marrow morphological findings. The top picture is 100× and the bottom is 10×. **(B)** Results of bone marrow biopsy. The test was performed using HE Gomori staining. **(C)** Immunophenotyping results of bone marrow flow cytometry. **(D)** Results of bone marrow chromosome examination. The test was performed using Giemsa banding.

On July 15, a 50-year-old woman experienced chest tightness, fatigue, dizziness, headache, and reduced endurance. Ten days later, she developed skin petechiae, purpura, bleeding gums, and nosebleeds. Upon admission to Peking University People’s Hospital, her blood test showed WBC 2.0 × 10^9^/L, absolute neutrophil count (ANC) 0.17 × 10^9^/L, hemoglobin (HBG) 42 g/L, PLT 2 × 10^9^/L, reticulocyte (RET) 0.071 × 10^9^/μL (normal range 0.024–0.084), and RET% 0.61 (normal range 0.5–1.5). Considering hematologic dystem diseases, aplastic anemia (AA) is highly likely. Blood transfusion, hemostasis and symptomatic supportive treatment were given, and the anemia symptoms improved after treatment. On August 14, bone marrow examination revealed markedly hypoproliferative morphology (< 25%), reduced granulopoiesis, decreased cells in all stages, erythroid suppression, few late erythroblasts, increased lymphocytes, absent dysplastic hematopoiesis, oil droplets and non-hematopoietic cell islands (Figure 1A). Biopsy revealed less than 5VOL% hematopoietic tissue volume, sparse erythroid and granulocytic hyperplasia, absent megakaryocytes, lymphocyte clusters, focal adipose tissue hyperplasia, and Gomori staining consistent with myelofibrosis (MF): MF-0 (Figure 1B). Immunophenotyping indicated elevated lymphocytes (88.71%), predominantly CD3 + T cells accounted for 79.76%, of which CD3+ TCRr/d- cells accounted for 79.26%, with a normal CD4/CD8 ratio (1.32) and normal TRBC1 expression (33.20%). CD3 + TCRr/d + cells were increased (20.31%) and characterized as CD16 + CD56part + CD57+. TCRr/d2+ cells represented 91.95% (Increased). CD19+ B cells accounted for 6.12%, mainly CD20+ mature B cells, with a normal Kappa/Lambda ratio of 1.60. Granulocytes was notably reduced (1.36%), with decreased CD10+ mature granulocytes (39.34%) and increased CD56+ cells (9.18%). Monocytes accounted for 0.60%, and CD34 + CD117+ immature myeloid cells were absent (Figure 1C). High-resolution chromosome analysis revealed a normal female karyotype, 46XX[20] (Figure 1D). Genetic testing and myeloid tumor-related next-generation sequencing were negative. Based on the above results, the patient was diagnosed with VSAA.

Subsequently, the patient received intermittent blood transfusion and hemostasis symptomatic supportive treatment at the outpatient clinic of Peking University People’s Hospital. During the outpatient period, the patient developed sore throat, cough, blood in sputum, and congestion of the posterior pharyngeal wall. Upper respiratory tract infection was considered, and cefaclor capsules (0.25 g, bid) were given for anti-infection treatment. Subsequently, the patient developed fever symptoms and elevated CRP. Ertapenem (1 g, once, ivdrip) was given intravenously for anti-infection and acetaminophen for antipyretic. However, CRP continued to rise, and enzyme-producing resistant bacteria infection was considered. On September 1, vancomycin (1 g, q12h) combined with imipenem (500 mg, q8h) for anti-infection treatment was added. During the treatment, CRP showed a gradual downward trend and the peak temperature decreased. On September 5, she was admitted to the Department of Hematology of Beijing Qinghe Hospital for inpatient treatment. She had no history of diabetes, hypertension, surgery, trauma, blood transfusion, liver disease, or tuberculosis exposure.

**Figure 2:**
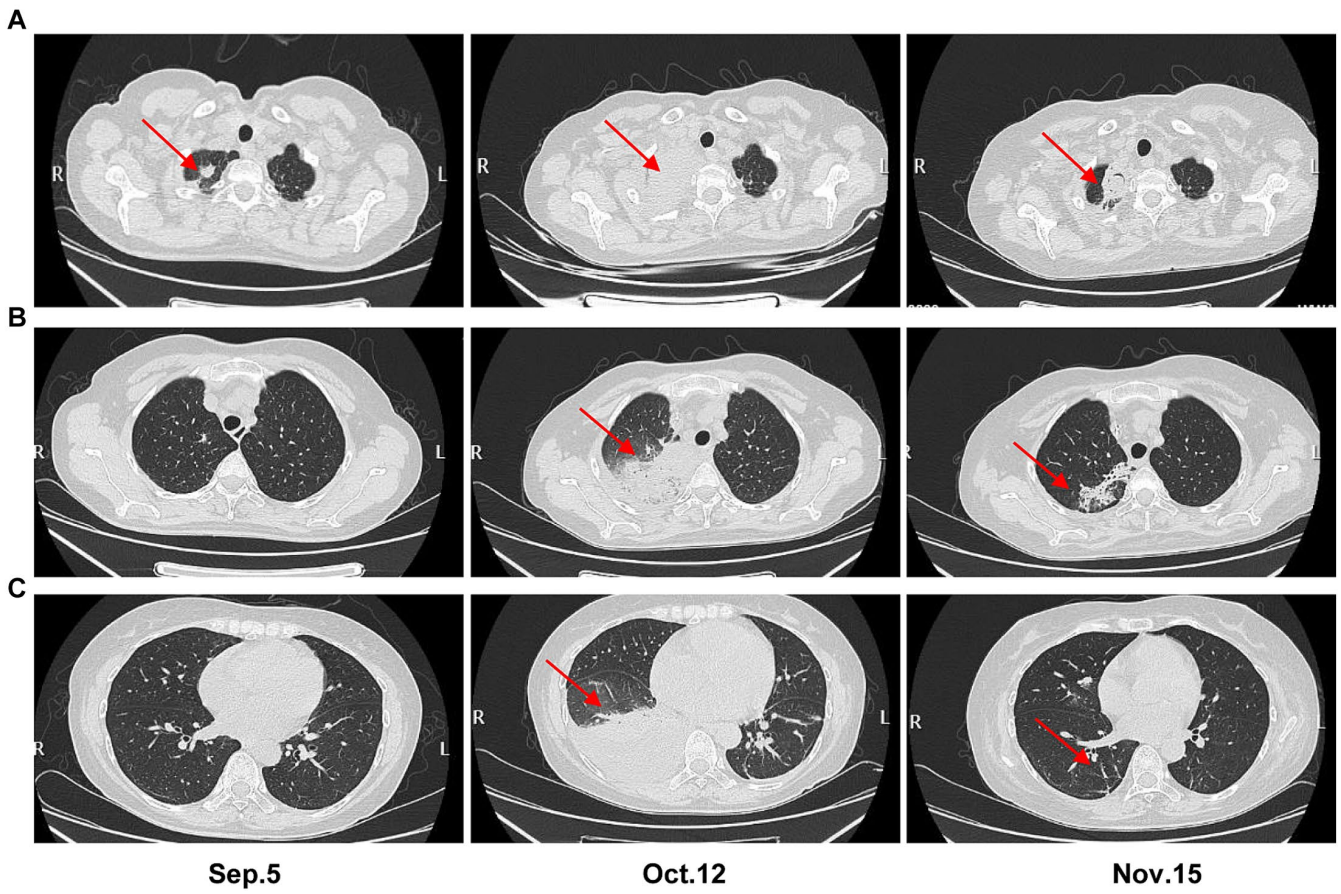
Chest CT scans at three time points (on admission, Oct.12, and Nov.15). **(A–C)** are the imaging findings of the apex of the right lung, the lower lobe of the right lung and the site of pleural effusion, respectively.

Upon admission, she presented with a low-grade fever (maximum 38°C), occasional cough without sputum production, and laboratory findings included WBC 1.29 × 10^9^/L, ANC 0.02 × 10^9^/L, HBG 65 g/L, PLT 32 × 10^9^/L, C-reactive protein (CRP) 45.25 mg/L, and aspartate aminotransferase (AST) 55 U/L. Tests for *Mycoplasma*, *Chlamydia*, cytomegalovirus (CMV), Epstein–Barr virus (EBV), and parvovirus were negative. Tests for 1,3-β-D-glucan (G) and galactomannan (GM) were also negative. The kit used for G and GM test were Fungal (1–3)-β-D-glucan detection kit (chromogenic method; Dynamiker Biotechnology, China) and *Aspergillus* galactomannan detection kit (enzyme-linked immunosorbent assay; Dynamiker Biotechnology, China), respectively. Chest computed tomography (CT) revealed a mass with patchy shadows in the apex of the right lung, irregular in shape with strip shadows around it. No abnormalities were noted in the lower right lung lobe, and there was no significant pleural effusion (Figure 2). Given the suspicion of fungal infection complicating pulmonary infection, the patient received meropenem (1 g, q8h, Sep.5–14) and caspofungin (70 mg for the first day, followed by 50 mg, ivdrip, qd, Sep. 5–28).

After 1 week of treatment, the patient continued to experience low-grade afternoon fever, which escalated to above 38.0°C on September 11. Concurrently, her CRP levels showed a steady increase. Peripheral blood samples were collected for mNGS testing, following established protocols (13, 15). DNA and RNA were extracted and prepared into sequencing libraries. Sequencing was conducted using the MGISEQ-200 platform (MGI Technology) with a 50 bp single-end sequencing kit. Raw FASTQ-format data underwent quality control using Fastq software. High-quality sequencing reads aligning with human reference genome GRCh37 (hg19) were removed with Bowtie2 v2.4.3, and the remaining sequences were annotated against the NCBI GenBank database using Kraken2 v2.1.0 to identify pathogens. Pathogens were identified based on the specific reads per 10 million value (RPTM value), with a threshold of ≥3 for viruses and ≥ 8 for bacteria and fungi. Results revealed 6 RPTM of Herpes simplex virus 1 (HSV-1) and 4 RPTM of *A. flavus*. Antiviral therapy with acyclovir (0.5 g, q12h, Sep.12–26) was initiated. By September 14, the patient’s fever peaked at 39.2°C with minimal sputum production. Empirical antibiotic adjustments were made. Antibiotics were changed to cefoperazone/sulbactam (3 g, q8h, Sep.14–22) in combination with tigecycline (100 mg, q12h during Sep.14–18, reduced to 50 mg, q12h during Sep.19–26), with concurrent human immunoglobulin infusion. The G and GM test were negative. On September 17, sputum culture showed carbapenem-resistant *Chryseobacterium indologenes*, sensitive to levofloxacin per drug susceptibility test (DST). After adding levofloxacin (100 mL, qd, Sep.18–26), the patient’s peak temperature decreased to 37.5°C, with decreased fever duration and CRP levels. However, fever recurred on the afternoon of September 20, reaching 38.9°C, with positive G (257.13 ng/L) and GM (0.66 μg/L) tests but negative blood culture. Subsequent sputum culture on September 22 identified *Pseudomonas aeruginosa*, sensitive to imipenem and meropenem as per DST. Blood mNGS detected *P. aeruginosa* (RPTM = 40) and *A. flavus* (RPTM = 69), with *A. flavus* reads showing a 17.25-fold increase from previous levels. Therapy was adjusted to include imipenem (0.5 g, q6h, Sep.22–28; based on DST result) and liposomal amphotericin B (from 25 mg to 100 mg, qd, Sep.22–27) for antifungal therapy, resulting in gradual normalization of the patient’s temperature.

**Figure 3:**
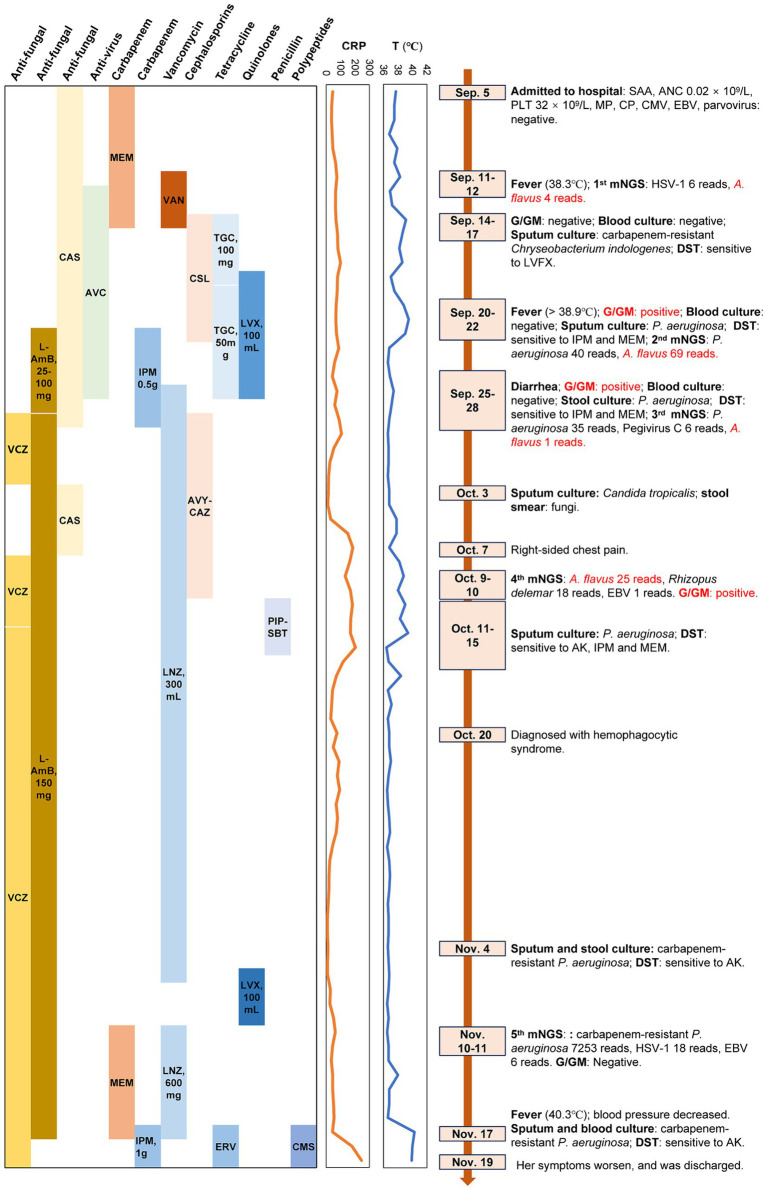
The clinical course of the patient (schematic). T, temperature; VSAA, Very severe aplastic anemia; ANC, Absolute Neutrophil Count; PLT, platelets; CMV, Cytomegalovirus; EBV, Epstein–barr virus; DST, Drug susceptibility testing; G/GM test, 1, 3-β-D-glucan/ galactomannan test; VCZ, voriconazole; L-AmB, liposomal amphotericin B; CAS, caspofungin; AVC, acyclovir; MEM, meropenem; IPM, imipenem; VAN, vancomycin; LNZ, linezolid; CLS, cefoperazone/sulbactam; AVY-CAZ, ceftazidime/avibactam; TGC, tigecycline; ERV, eravacycline; LVX, levofloxacin; PIP-SBT, piperacillin and sulbactam; CMS, colistimethate sodium; RPTM, reads per 10 million.

On September 25, the patient developed diarrhea, prompting a stool culture that tested positive for *P. aeruginosa*, with DST showing sensitivity to imipenem and meropenem by September 27. Blood culture was negative, while the G (88.11 ng/L) and GM (2.09 μg/L) tests remained positive. The third blood mNGS detected *P. aeruginosa* (RPTM = 35), Pegivirus C (RPTM = 6), and low levels of *A. flavus* (RPTM = 1), suggesting efficacy of the current antifungal therapy. Voriconazole (0.2 g, q12h, Sep.28-Oct.2) and caspofungin (50 mg, qd, Oct.3–7) were alternated with ongoing liposomal amphotericin B (150 mg, qd, Sep.28-Nov.17) for antifungal coverage. Imipenem was discontinued (due to diarrhea and a increased CRP) and replaced by ceftazidime avibactam sodium (2.5 g, q8h, Sep.28-Oct.10) in combination with linezolid (300 mg, q12h, Sep.26-Nov.6; the change of this was because the patient developed gastrointestinal intolerance such as nausea and poor appetite with using tigecycline). On October 3, sputum culture revealed *Candida tropicalis*, confirmed by fungal elements in stool smear. Four days later, the patient experienced right chest pain. By October 10, the fourth blood mNGS reported *A. flavus* (RPTM = 25), *Rhizopus delemar* (RPTM = 18), and EBV (RPTM = 1). Concurrently, positive G (114.1 ng/L) and GM (1.15 μg/L) tests results were noted. *P. aeruginosa* was identified in sputum culture, sensitive to amikacin, imipenem and meropenem. Chest CT imaging showed worsening infection in the right lung apex, increased consolidation in the lower right lung lobe, and bilateral pleural effusion, more prominent on the right side (Figure 2). Intravenous voriconazole (0.2 g, q12h, Oct.13-Nov.19) and continued liposomal amphotericin B were initiated. The fifth blood mNGS on November 10, identified carbapenem-resistant *P. aeruginosa* (OXA-50), consistent with the sputum culture findings, along with 6 RPTM of EBV and 18 RPTM of HSV-1; no *A. flavus* was identified. G/GM tests were negative. Subsequent chest CT scan showed cavity formation in the right lung apex due to resolving infection, decreased consolidation in the lower lung lobe, and complete resolution of pleural effusion (Figure 2). Based on etiological and imaging findings, significant improvement in pulmonary fungal and bloodstream infections were observed. Figure 3 displays the patient’s symptoms, etiologic test results, and disease progression over time. The results of procalcitonin (PCT), WBC, ANC, PLT, and HBG during hospitalization were showed in Supplementary Figure 1.

Throughout treatment, the patient underwent five blood mNGS tests, with the first four showing persistent *A. flavus* positive despite antifungal treatment, highlighting the challenges in clearing *A. flavus* infection in immunocompromised SAA patients. The fifth test showed *A. flavus* negativity, indicating the efficacy of continuous use of liposomal amphotericin B and voriconazole therapy against *Aspergillus* infection. Blood and sputum culture were negative, and the G/GM test results were consistent with the mNGS findings. The presence of neutropenia with GM > 1.0, in conjunction with cavity or consolidation observed on chest CT, could indicate the presence of an probable invasive fungal infection in the patient (16).

## 3 Discussion

*A. flavus* is the second most common pathogenic species causing invasive *Aspergillus* infections, account for approximately 15–20% of cases (6, 17). Invasive *Aspergillus* infection usually occurs in conditions involving significant defects in cellular phagocytosis, such as severe and prolonged neutropenia (e.g., after hematopoietic stem-cell transplantation or aplastic anemia), and in patients undergoing high-dose glucocorticoid therapy or those with hematologic malignancies or chronic granulomatous disease (6). While the lung is the most affected organ by invasive aspergillosis, hematogenous spread can involve internal organs, particularly the central nervous system and the skin. Classic clinical symptoms include fever, chest pain, and hemoptysis, with lung imaging often showing patchy infiltrates or nodules that may cavitate, along with macronodules or patchy consolidation (18). In a Tunisian study, *A. flavus* was isolated from invasive lung disease in 73.7% of neutropenic patients, among whom cough, chest pain, and hemoptysis were predominant symptoms. CT scans consistently showed cavitated lesions and nodules (19). In our study, fever appeared early and persisted, while chest pain developed in the intermediated to advanced stages. CT findings revealed nodules and patchy shadows, which are crucial for diagnosing *Aspergillus* infection.

Culture remains the gold standard for pathogen identification, yet it is time-consuming and has limited sensitivity (20). The G and GM tests serve as diagnostic tools for suspected IPA (21). However, the sensitivity and specificity of these tests vary among studies, with higher specificity observed in high-risk patients (22, 23), but lower sensitivity noted in patients lacking pre-existing neutropenia (24, 25). GM results ≥1.0, positive *Aspergillus* PCR, and positive respiratory sample culture are considered mycological evidence of *Aspergillus* infection (16). In our study, fungal infection diagnosis was supported by positive G/GM tests and multiple mNGS results. Compared to the CMT methods for fungi detection, the advantages of mNGS including higher sensitivity, detected fungal positivity earlier than the G/GM tests (7–9), and were less affected by antibiotics (26, 27). Nevertheless, in order to prevent false-positive mNGS results, multiple or multiple sites need to be sent for testing or other fungal-related tests to verify the mNGS results. Early pathogen detection not only reduces antibiotics misuse, but also effectively hinders disease progression. Moreover, multiple mNGS tests in the same individual can capture longitudinal dynamic changes in viral load among transplant patients (28, 29), aiding in determining optimal sampling times for patients with bloodstream infections (30–32). Our findings underscore the role of multiple mNGS tests in promptly identifying infection etiology when clinical etiology tests are negative. These results also highlight the importance of performing multiple mNGS tests not only to verify each other and identify the cause as quickly as possible, but also to monitor the patient’s infection progression status.

Currently, *Aspergillus* infections in patients with AA are rare. We conducted a comprehensive literature search in the Web of Science and PubMed databases using the keywords “*Aspergillus*,” “Aplastic anemia,” and “Pulmonary infection” up to April 2024. Only studies that included detailed case information on adult patients with aplastic anemia and pulmonary *Aspergillus* infection were included. A total of 7 cases were obtained involving patients aged 18 years or older (Table 1), summarized chronologically by publication date. Among the seven patients, four were male and three were female, with ages ranging from 18 to 75 years (median: 23 years). Five patients presented with fever, and three experienced coughing. Pulmonary imaging commonly revealed infiltration (71.43%). Culture was predominantly used for pathogen identification, with four patients infected by *A. fumigatus*, one by *A. viridinutans*, one with unidentified *Aspergillus* species, and one with negative etiological findings but positive response to antifungal therapy. To the best of our knowledge, this case represents the first reported case of *A. flavus* infection in VSAA, contributing significantly to the understanding of *Aspergillus* infection associated with AA. Fever was also the main symptoms for this patient, consolidation and pleural effusion were the main features of the chest CT results.

**Table 1:** Clinical information of aplastic anemia patients infected with *Aspergillus*.

Reference	Publication year	Country/year	Age/gender	Clinical presentation	Imaging	Pathogen	Infection site	Diagnostic methods^a^	Treatment	Transplant	Prognosis
1 (56)	2021	Germany/2018	59/Male	A sudden rise in serum inflammatory markers and a strongly positive GM assay	CT: a diffuse infiltrate of alveolar and perironchial tissue	*A. fumigatus; C. glabrata*	pulmonary	BALF culture	NA	Yes	Death
2 (57)	2017	Austria/2016	23/Male	Petechial bleeding, with haematoma on the right cubita, chills and fever	CT: three subpleural nodules up to 28 mm in diameter	negative	pulmonary	GM, culture, and PCR	posaconazole, Caspofungin, isavuconazole	Yes	Survival
3 (58)	2014	Japan/2014	75/Female	Fever, cough	CT: a mass shadow with a central low attenuation area in the lower lobe of the left lung	*A. viridinutans*	pulmonary	BALF culture, PAS and GMS stain	Caspofungin and Voriconazole	No	Survival
4 (59)	2006	Japan/2000	38/Male	Fever, cough, anterior chest pain	Chest x-ray: pulmonary infiltration of the left lower lobe	*Aspergillus*	pulmonary, brain and heart	serum *Aspergillus* antigen, GM, GMS stain	amphotericin B	No	Death
5 (60)	2002	Japan/1999	19/Female	fever, cough, and stridor, inspiratory dyspnea	Chest x-ray: pulmonary infiltration	*A. fumigatus*	pulmonary	culture	Nebulized amphotericin B	No	Death
6 (61)	1997	Turkey/1997	18/Female	Fever, weakness and bluish spots in the skin, headache	Chest x-ray: an infiltrate in the lower segment of the upper right lobe	*A. fumigatus*	pulmonary, brain	Pus culture	Liposomal amphotericin B and itraconazole	Yes	Survival
7 (62)	1991	Belgium/1991	21/Male	Severe pain in the right shoulder and an infiltrate in the right lung top	CT: a necrotizing pneumonia of the right lung top with infiltration of the thoracic wall	*A. fumigatus*	pulmonary, soft tissue	Sputum and soft tissue culture	amphotericin B, ambisome, itraconazole	No	Survival

a GMS, Grocott-Gomori methenamine-silver nitrate stain; PAS, Periodic acid-Schiff stain; BALF, Bronchoalveolar lavage fluid; CT, Computed Tomography.

Voriconazole is the preferred treatment for invasive fungal disease (IFD) (25, 33, 34), while isavuconazole and posaconazole are less effective (35, 36). Guidelines strongly recommend liposomal amphotericin B (L-AmB) as an initial alternative treatment for IFD, typically at 3 mg/kg/day (25, 33, 34). Initial high-dose L-AmB (10 mg/kg/day) does not offer superior efficacy over standard dose (37). Treatment duration typically ranges from 6 to 12 weeks, depending on disease severity, symptom resolution, and immune recovery (38). Posaconazole, fluconazole, micafungin, caspofungin or voriconazole are mainly used for primary prevention in China, with European guidelines recommending nebulized inhalation of 10 mg of L-AmB twice weekly in combination with fluconazole for patients undergoing initial induction chemotherapy. Furthermore, the multidisciplinary expert consensus on the clinical rational application of different formulations of amphotericin B (2024 edition) (39) points out: For patients with high risk of monotherapy failure, multi-site infection or drug-resistant IFD, combination therapy with two drugs can be considered to improve efficacy and survival rate, such as L-AmB + echinocandin drugs, L-AmB + voriconazole and other triazole anti-aspergillus drugs (including voriconazole and other triazole anti-aspergillus drugs + echinocandin combination therapy). A review of the literature revealed that approximately eight studies evaluated the therapeutic efficacy of AmB combined with voriconazole in IFD. Two of these studies were *in vitro* observations (40, 41), three were in animal studies (42–44), while the final three were patient observations (45–47). The *in vitro* observations demonstrated that combinations of antifungal therapy exhibited promising results. One animal observation indicated that the efficacy of monotherapy and two-drug combination therapy was comparable, with no discernible enhancement or reduction in activity associated with the latter (42). Two additional animal studies demonstrated that the combination of AmB and voriconazole was more effective than either agent alone in reducing tissue burden, sterilizing tissues, and reducing mortality (43, 44). One observational study in humans indicated that a triple combination of AmB, caspofungin, and voriconazole may be a promising avenue for further investigation in patients with refractory fungal infections who have not responded to more conventional therapies (47). Another two patient observations demonstrated that AmB combined with voriconazole may rapidly improve clinical manifestations and substantially shorten hospitalization times (45, 46). Among the seven cases reviewed in our study, AmB/L-AmB was the most frequently administered drug (57.14%; Table 1). In this study, the patient weighted 46 kg. Initially, caspofungin (70 mg for the first day, then 50 mg, ivdrip, qd) was used for antifungal therapy when fungal infection was suspected, but lung symptoms did not improve. After the second mNGS confirmed *A. flavus* positivity, L-AmB (from 25 mg to 100 mg, qd) antifungal treatment was added, leading to gradual normalization of body temperature. Continuous detection of *A. flavus* by the third mNGS and positive G/GM tests prompted continued use L-AmB (150 mg, qd), alongside alternate use of voriconazole (0.2 g, q12h) and caspofungin (50 mg, qd). Subsequent mNGS showed persistent *A. flavus* presence alongside positive G/GM tests, prompting intravenous voriconazole (0.2 g, q12h) combined with L-AmB (increased to 200 mg qd for 5 days, then reduced to the original 150 mg qd due to increased creatinine levels, indicating renal function changes). The fifth mNGS test was negative for *A. flavus*, with negative G/GM test results, and improved pulmonary symptoms, indicating effective antifungal therapy. The above results show that L-AmB combined with voriconazole is effective in treating severe invasive fungal infections, but the dosage of L-AmB should be controlled to prevent renal damage in patients.

In addition to *A. flavus*, attention must be paid to carbapenem-resistant *Pseudomonas aeruginosa* (CRPA) as a late-stage pathogen in this case. *P. aeruginosa* has multiple mechanisms of drug resistance, often exacerbated by frequent antibiotic use (48). Patients with hematological disorders, including VSAA (49), who acquire infections caused by drug-resistant *P. aeruginosa* faced elevated mortality rates (50, 51). In our study, *P. aeruginosa* was initially detected with low read numbers (RPTM = 40 and 35) in the 2nd and 3rd mNGS tests but was absent in the 4th test. However, 1 month later, the 5th mNGS detected drug-resistant *P. aeruginosa* (OXA-50) with RPTM was 7,253. In addition, herpesviruses are also potential pathogens in AA patients (4, 52), significantly impacting mortality rates, particularly in severely ill or immunocompromised individuals (53–55). In our study, HSV-1 was identified in the 1st mNGS, and acyclovir was administered for 15 days to control the infection, after which HSV-1 did not recur. However, after a 13-day interval, another herpesvirus, EBV, appeared in the 4th mNGS test and increased in abundance in the 5th mNGS results, alongside recurrent HSV-1 detection. The intermittent presence of herpesviruses and the emergence of CRPA suggest a challenging prognosis for this patient. These results underscore the importance of monitoring and preventing drug resistance in immunocompromised patients with bloodstream infection, while also emphasizing the significance herpesviruses surveillance.

This study describes a rare case of *A. flavus* aplastic anemia in which invasive fungal infection was confirmed by multiple mNGS tests despite negative clinical etiology test results. This case highlights the important diagnostic value and medication guidance value of mNGS testing for severe invasive *Aspergillus* infection, and also demonstrates the therapeutic effect of L-AmB combined with voriconazole. However, this study also has shortcomings. A single case has little significance for medication guidance. Subsequent cohort studies are needed to further prove the diagnostic effect of L-AmB combined with voriconazole on severe invasive *Aspergillus* infection.

## 4 Conclusion

In summary, we used mNGS to diagnose a case of VSAA caused by *A. flavus* infection, effectively managed with a combination of voriconazole and liposomal amphotericin B. This demonstrate that amphotericin B combined with voriconazole is an effective treatment strategy for patients with severe invasive fungal infections. This case demonstrates that in patients with long-term immunocompromised states due to a granulocytopenia, timely and iterative mNGS testing can promptly identify infection status when clinical routine microbiological tests fail to ascertain the disease etiology. Such insights are crucial for early detection, accurate diagnosis, and treatment planning.

## Data Availability

The raw data supporting the conclusions of this article will be made available by the authors, without undue reservation.

## References

[1] Killick SB Bown N Cavenagh J Dokal I Foukaneli T Hill A. Guidelines for the diagnosis and management of adult aplastic anaemia. Br J Haematol. (2015) 172:187–207. doi: 10.1111/bjh.13853 26568159

[2] Valdez JM Scheinberg P Young NS Walsh TJ. Infections in patients with aplastic Anemia. Semin Hematol. (2009) 46:269–76. doi: 10.1053/j.seminhematol.2009.03.008 19549579

[3] Marsh JCW Ball SE Cavenagh J Darbyshire P Dokal I Gordon-Smith EC. Guidelines for the diagnosis and management of aplastic anaemia. Br J Haematol. (2009) 147:43–70. doi: 10.1111/j.1365-2141.2009.07842.x 19673883

[4] Li Y Xiong Y Fan H Jing L Li J Lin Q. Metagenomic next-generation sequencing of plasma for the identification of bloodstream infectious pathogens in severe aplastic anemia. Chinese J Hematol. (2023) 44:236–41. doi: 10.3760/cma.j.issn.0253-2727.2023.03.010 PMC10119722 37356986

[5] Lian X Scott-Thomas A Lewis JG Bhatia M Macpherson SA Zeng Y. Monoclonal antibodies and invasive aspergillosis: diagnostic and therapeutic perspectives. Int J Mol Sci. (2022) 23:5563. doi: 10.3390/ijms23105563 35628374 PMC9146623

[6] Rudramurthy SM Paul RA Chakrabarti A Mouton JW Meis JF. Invasive aspergillosis by aspergillus flavus: epidemiology, diagnosis, antifungal resistance, and management. J Fungi. (2019) 5:55. doi: 10.3390/jof5030055 PMC6787648 31266196

[7] Hill JA Dalai SC Hong DK Ahmed AA Ho C Hollemon D. Liquid biopsy for invasive Mold infections in hematopoietic cell transplant recipients with pneumonia through next-generation sequencing of microbial cell-free DNA in plasma. Clin Infect Dis. (2021) 73:e3876–83. doi: 10.1093/cid/ciaa1639, PMID: 33119063 PMC8664431

[8] Hoenigl M Egger M Price J Krause R Prattes J White PL. Metagenomic next-generation sequencing of plasma for diagnosis of COVID-19-associated pulmonary aspergillosis. J Clin Microbiol. (2023) 61:e0185922. doi: 10.1128/jcm.01859-22 36809121 PMC10035327

[9] Yang L Song J Wang Y Feng J. Metagenomic next-generation sequencing for pulmonary fungal infection diagnosis: lung biopsy versus Bronchoalveolar lavage fluid. Infect Drug Resist. (2021) 14:4333–59. doi: 10.2147/IDR.S333818 34707378 PMC8542593

[10] Chen H Yin Y Gao H Guo Y Dong Z Wang X. Clinical utility of in-house metagenomic next-generation sequencing for the diagnosis of lower respiratory tract infections and analysis of the host immune response. Clin Infect Dis. (2020) 71:S416–26. doi: 10.1093/cid/ciaa1516 33367583

[11] Zanella MC Cordey S Laubscher F Docquier M Vieille G Van Delden C. Unmasking viral sequences by metagenomic next-generation sequencing in adult human blood samples during steroid-refractory/dependent graft-versus-host disease. Microbiome. (2021) 9:28. doi: 10.1186/s40168-020-00953-3 33487167 PMC7831233

[12] Cheng J Hu H Fang W Shi D Liang C Sun Y. Detection of pathogens from resected heart valves of patients with infective endocarditis by next-generation sequencing. Int J Infect Dis. (2019) 83:148–53. doi: 10.1016/j.ijid.2019.03.007 30926543

[13] Wu C Yu X Gai W Liu Y Qi Y Zheng Y. Diagnostic value of plasma and blood cells metagenomic next-generation sequencing in patients with sepsis. Biochem Biophys Res Commun. (2023) 683:149079. doi: 10.1016/j.bbrc.2023.10.011 37871447

[14] Camitta BM Rappeport JM Parkman R Nathan DG. Selection of patients for bone marrow transplantation in severe aplastic anemia. Blood. (1975) 45:355–63. 1090310

[15] Chen H Zheng Y Zhang X Liu S Yin Y Guo Y. Clinical evaluation of cell-free and cellular metagenomic next-generation sequencing of infected body fluids. J Adv Res. (2023) 55:119–29. doi: 10.1016/j.jare.2023.02.018 36889461 PMC10770109

[16] Donnelly JP Chen SC Kauffman CA Steinbach WJ Baddley JW Verweij PE. Revision and update of the consensus definitions of invasive fungal disease from the European Organization for Research and Treatment of Cancer and the mycoses study group education and research consortium. Clin Infect Dis. (2020) 71:1367–76. doi: 10.1093/cid/ciz1008 31802125 PMC7486838

[17] Krishnan S Manavathu EK Chandrasekar PH. Aspergillus flavus: an emerging non-fumigatus aspergillus species of significance. Mycoses. (2009) 52:206–22. doi: 10.1111/j.1439-0507.2008.01642.x 19207851

[18] Rj J Jn P Js B. Opportunistic bacterial, viral and fungal infections of the lung. Medicine (Abingdon). (2020) 48:366–72. doi: 10.1016/j.mpmed.2020.03.006 32390758 PMC7206443

[19] Saghrouni F Ben Youssef Y Gheith S Bouabid Z Ben Abdeljelil J Khammari I. Twenty-nine cases of invasive aspergillosis in neutropenic patients. Med Mal Infect. (2011) 41:657–62. doi: 10.1016/j.medmal.2011.09.011, PMID: 22036518

[20] Simner PJ Miller S Carroll KC. Understanding the promises and hurdles of metagenomic next-generation sequencing as a diagnostic tool for infectious diseases. Clin Infect Dis. (2018) 66:778–88. doi: 10.1093/cid/cix881 29040428 PMC7108102

[21] Lehrnbecher T Hassler A Groll AH Bochennek K. Diagnostic approaches for invasive aspergillosis-specific considerations in the pediatric population. Front Microbiol. (2018) 9:518. doi: 10.3389/fmicb.2018.00518 29632518 PMC5879093

[22] Lehrnbecher T Robinson PD Fisher BT Castagnola E Groll AH Steinbach WJ. Galactomannan, β-D-glucan, and polymerase chain reaction-based assays for the diagnosis of invasive fungal disease in pediatric Cancer and hematopoietic stem cell transplantation: a systematic review and Meta-analysis. Clin Infect Dis. (2016) 63:1340–8. doi: 10.1093/cid/ciw592, PMID: 27567122

[23] Yeoh DK Mcmullan BJ Clark JE Slavin MA Haeusler GM Blyth CC. The challenge of diagnosing invasive pulmonary aspergillosis in children: a review of existing and emerging tools. Mycopathologia. (2023) 188:731–43. doi: 10.1007/s11046-023-00714-4 37040020 PMC10564821

[24] Huppler AR Fisher BT Lehrnbecher T Walsh TJ Steinbach WJ. Role of molecular biomarkers in the diagnosis of invasive fungal diseases in children. J Pediatric Infect Dis Society. (2017) 6:S32–44. doi: 10.1093/jpids/pix054 PMC5907877 28927202

[25] Patterson TF Thompson GR 3rd Denning DW Fishman JA Hadley S Herbrecht R. Practice guidelines for the diagnosis and Management of Aspergillosis: 2016 update by the Infectious Diseases Society of America. Clin Infect Dis. (2016) 63:e1–e60. doi: 10.1093/cid/ciw326 27365388 PMC4967602

[26] Liang W Zhang Q Qian Q Wang M Ding Y Zhou J. Diagnostic strategy of metagenomic next-generation sequencing for gram negative bacteria in respiratory infections. Ann Clin Microbiol Antimicrob. (2024) 23:10. doi: 10.1186/s12941-024-00670-x. 38302964 PMC10835912

[27] Wu Y Wu J Xu N Lin M Yue W Chen Y. Clinical application value of metagenome next-generation sequencing in pulmonary diffuse exudative lesions: a retrospective study. Front Cell Infect Microbiol. (2024) 14:1367885. doi: 10.3389/fcimb.2024.1367885 38784566 PMC11113015

[28] Bal A Sarkozy C Josset L Cheynet V Oriol G Becker J. Metagenomic next-generation sequencing reveals individual composition and dynamics of Anelloviruses during autologous stem cell transplant recipient management. Viruses. (2018) 10:633. doi: 10.3390/v10110633 30441786 PMC6266913

[29] Carbo E Russcher A Kraakman M De Brouwer C Sidorov I Feltkamp M. Longitudinal monitoring of DNA viral loads in transplant patients using quantitative metagenomic next-generation sequencing. Pathogens. (2022) 11:236. doi: 10.3390/pathogens11020236, PMID: 35215180 PMC8874692

[30] Gyarmati P Kjellander C Aust C Song Y Ohrmalm L Giske CG. Metagenomic analysis of bloodstream infections in patients with acute leukemia and therapy-induced neutropenia. Sci Rep. (2016) 6:23532. doi: 10.1038/srep23532 26996149 PMC4800731

[31] Qin C Zhang S Zhao Y Ding X Yang F Zhao Y. Diagnostic value of metagenomic next-generation sequencing in sepsis and bloodstream infection. Front Cell Infect Microbiol. (2023) 13:1117987. doi: 10.3389/fcimb.2023.1117987 36844396 PMC9950395

[32] Vijayvargiya P Feri A Mairey M Rouillon C Jeraldo PR Esquer Garrigos Z. Metagenomic shotgun sequencing of blood to identify bacteria and viruses in leukemic febrile neutropenia. PLoS One. (2022) 17:e0269405. doi: 10.1371/journal.pone.0269405 35709201 PMC9202879

[33] Tissot F Agrawal S Pagano L Petrikkos G Groll AH Skiada A. ECIL-6 guidelines for the treatment of invasive candidiasis, aspergillosis and mucormycosis in leukemia and hematopoietic stem cell transplant patients. Haematologica. (2017) 102:433–44. doi: 10.3324/haematol.2016.152900 28011902 PMC5394968

[34] Ullmann AJ Aguado JM Arikan-Akdagli S Denning DW Groll AH Lagrou K. Diagnosis and management of aspergillus diseases: executive summary of the 2017 ESCMID-ECMM-ERS guideline. Clin Microbiol Infect. (2018) 24:e1–e38. doi: 10.1016/j.cmi.2018.01.002 29544767

[35] Maertens JA Raad I Marr KA Patterson TF Kontoyiannis DP Cornely OA. Isavuconazole versus voriconazole for primary treatment of invasive mould disease caused by aspergillus and other filamentous fungi (SECURE): a phase 3, randomised-controlled, non-inferiority trial. Lancet. (2016) 387:760–9. doi: 10.1016/s0140-6736(15)01159-9 26684607

[36] Maertens JA Rahav G Lee DG Ponce-De-León A Ramírez Sánchez IC Klimko N. Posaconazole versus voriconazole for primary treatment of invasive aspergillosis: a phase 3, randomised, controlled, non-inferiority trial. Lancet. (2021) 397:499–509. doi: 10.1016/S0140-6736(21)00219-1, PMID: 33549194

[37] Lass-Flörl C. Standard dosing regimen of liposomal amphotericin B is as effective as a high-loading dose for patients with invasive aspergillosis: AmBiLoad trial. Expert Rev Anti-Infect Ther. (2007) 5:929–32. doi: 10.1586/14787210.5.6.929 18039077

[38] Chinese-Association-Hematologists, and Chinese-Invasive-Fungal-Infection-Working-Group. The Chinese guidelines for the diagnosis and treatment of invasive fungal disease in patients with hematological disorders and cancers (the 6th revision). Chinese J Internal Med. (2020) 59:754–63. doi: 10.3760/cma.j.cn112138-20200627-00624 32987477

[39] Medical-Mycology-Society-of-Chinese-Medicin-and-Education-Association. Multidisciplinary expert consensus on the clinical rational application of different formulations of amphotericin B. Chinese J Internal Med. (2024) 63:230–57. doi: 10.3760/cma.j.cn112138-20231122-00332 38448188

[40] O'shaughnessy EM Meletiadis J Stergiopoulou T Demchok JP Walsh TJ. Antifungal interactions within the triple combination of amphotericin B, caspofungin and voriconazole against aspergillus species. J Antimicrob Chemother. (2006) 58:1168–76. doi: 10.1093/jac/dkl392 17071635

[41] Oz Y Kiremitci A Dag I Metintas S Kiraz N. Postantifungal effect of the combination of caspofungin with voriconazole and amphotericin B against clinical *Candida krusei* isolates. Med Mycol. (2013) 51:60–5. doi: 10.3109/13693786.2012.697198 22746405

[42] Chandrasekar PH Cutright JL Manavathu EK. Efficacy of voriconazole plus amphotericin B or micafungin in a guinea-pig model of invasive pulmonary aspergillosis. Clin Microbiol Infect. (2004) 10:925–8. doi: 10.1111/j.1469-0691.2004.00958.x 15373889

[43] Kirkpatrick WR Coco BJ Patterson TF. Sequential or combination antifungal therapy with voriconazole and liposomal amphotericin B in a Guinea pig model of invasive aspergillosis. Antimicrob Agents Chemother. (2006) 50:1567–9. doi: 10.1128/aac.50.4.1567-1569.2006 16569887 PMC1426916

[44] Silva EG Paula CR Dias AL Chang MR Ruiz Lda S Gambale V. Combination efficacy of voriconazole and amphotericin B in the experimental disease in immunodeficient mice caused by fluconazole-resistant *Cryptococcus neoformans*. Mycopathologia. (2011) 171:261–6. doi: 10.1007/s11046-010-9375-5 20972836

[45] Fujioka K Nagai T Kinoshita Y Urushihara M Hamasaki Y Shishido S. Successful treatment with voriconazole combined with amphotericin B-liposome for fluconazole-resistant pulmonary cryptococcosis after renal transplantation. CEN Case Rep. (2019) 8:261–5. doi: 10.1007/s13730-019-00403-6 31161376 PMC6820654

[46] Liu J Liu J Su X Yang L Wang Y Wang A. Amphotericin B plus fluorocytosine combined with voriconazole for the treatment of non-HIV and non-transplant-associated cryptococcal meningitis: a retrospective study. BMC Neurol. (2022) 22:274. doi: 10.1186/s12883-022-02803-1 35869441 PMC9306087

[47] Verma A Williams S Trifilio S Zembower T Mehta J. Successful treatment of concomitant pulmonary zygomycosis and aspergillosis with a combination of amphotericin B lipid complex, caspofungin, and voriconazole in a patient on immunosuppression for chronic graft-versus-host disease. Bone Marrow Transplant. (2004) 33:1065–6. doi: 10.1038/sj.bmt.1704485 15048146

[48] Pachori P Gothalwal R Gandhi P. Emergence of antibiotic resistance *Pseudomonas aeruginosa* in intensive care unit; a critical review. Genes Dis. (2019) 6:109–19. doi: 10.1016/j.gendis.2019.04.001 31194018 PMC6545445

[49] Lai S-Y Liu F Chang L Che G-L Yang Q-X Jiang Y-M. Multisite *Pseudomonas aeruginosa* infections detected by metagenomic next-generation sequencing in a child with aplastic Anemia: a case report. Lab Med. (2022) 53:e123–5. doi: 10.1093/labmed/lmab123 35075476

[50] Zhao Y Lin Q Liu L Ma R Chen J Shen Y. Risk factors and outcomes of antibiotic-resistant *Pseudomonas aeruginosa* bloodstream infection in adult patients with acute leukemia. Clin Infect Dis. (2020) 71:S386–93. doi: 10.1093/cid/ciaa1522 33367574

[51] Zhen S Zhao Y Chen Z Zhang T Wang J Jiang E. Assessment of mortality-related risk factors and effective antimicrobial regimens for treatment of bloodstream infections caused by carbapenem-resistant *Pseudomonas aeruginosa* in patients with hematological diseases. Front Cell Infect Microbiol. (2023) 13:1156651. doi: 10.3389/fcimb.2023.1156651 37415825 PMC10320591

[52] Levy I Laor R Jiries N Bejar J Polliack A Tadmor T. Amegakaryocytic thrombocytopenia and subsequent aplastic Anemia associated with apparent Epstein-Barr virus infection. Acta Haematol. (2018) 139:7–11. doi: 10.1159/000484595, PMID: 29301129

[53] Gatto I Biagioni E Coloretti I Farinelli C Avoni C Caciagli V. Cytomegalovirus blood reactivation in COVID-19 critically ill patients: risk factors and impact on mortality. Intensive Care Med. (2022) 48:706–13. doi: 10.1007/s00134-022-06716-y, PMID: 35583676 PMC9116062

[54] Huang L Zhang X Pang L Sheng P Wang Y Yang F. Viral reactivation in the lungs of patients with severe pneumonia is associated with increased mortality, a multicenter, retrospective study. J Med Virol. (2023) 95:e28337. doi: 10.1002/jmv.28337 36418241 PMC10099828

[55] Pan L Wu F Cai Q Xu Z Hu H Tang T. Whole genome profiling of lung microbiome in solid organ transplant recipients reveals virus involved microecology may worsen prognosis. Front Cell Infect Microbiol. (2022) 12:863399. doi: 10.3389/fcimb.2022.863399 35372133 PMC8967177

[56] Rejeski K Kunz WG Rudelius M Bücklein V Blumenberg V Schmidt C. Severe Candida glabrata pancolitis and fatal *Aspergillus fumigatus* pulmonary infection in the setting of bone marrow aplasia after CD19-directed CAR T-cell therapy – a case report. BMC Infect Dis. (2021) 21:121. doi: 10.1186/s12879-020-05755-4 33509115 PMC7841988

[57] Hoenigl M Prattes J Neumeister P Wölfler A Krause R. Real-world challenges and unmet needs in the diagnosis and treatment of suspected invasive pulmonary aspergillosis in patients with haematological diseases: an illustrative case study. Mycoses. (2017) 61:201–5. doi: 10.1111/myc.12727 29112326

[58] Kitaura T Chikumi H Murota H Fujiwara H Touge H Okada K. A case of lung abscess due to aspergillus viridinutans in a patient with aplastic anemia. Kansenshogaku Zasshi. (2014) 88:855–60. doi: 10.11150/kansenshogakuzasshi.88.855 25764808

[59] Itoh M Takahashi M Mori M Tamekiyo H Yoshida H Yago K. Myocardial infarction caused by aspergillus embolization in a patient with aplastic Anemia. Intern Med. (2006) 45:547–50. doi: 10.2169/internalmedicine.45.1607 16702749

[60] Nagasawa M Itoh S Tomizawa D Kajiwara M Sugimoto T Kumagai J. Invasive subglottal aspergillosis in a patient with severe aplastic Anemia: a case report. J Inf Secur. (2002) 44:198–201. doi: 10.1053/jinf.2001.0935 12099752

[61] Başlar Z Soysal T Hanci M Aygün G Ferhanoğlu B Sarioğlu AC. Successful outcome of aspergillus brain abscess in a patient who underwent bone marrow transplantation for aplastic anemia. Haematologia (Budap). (1997) 28:265–71. PMID: 9408772

[62] Bock RD Schrijvers D Peetermans M. Pulmonary aspergillosis complicated by osteomyelitis. Acta Clin Belg. (1991) 46:397–400. doi: 10.1080/17843286.1991.11718196 1665943

