# Supplementary material for: Rapid diagnosis of Aspergillus flavus infection in acute very severe aplastic anemia with metagenomic next-generation sequencing: a case report and literature review
Source: Front Med (Lausanne). 2024 Sep 23;11:1413964. doi: 10.3389/fmed.2024.1413964 (PMC11456449; doi:10.3389/fmed.2024.1413964)
Supplement: Supplementary file 1 [file Data_Sheet_1.docx]

Supplementary Material

# Supplementary Data


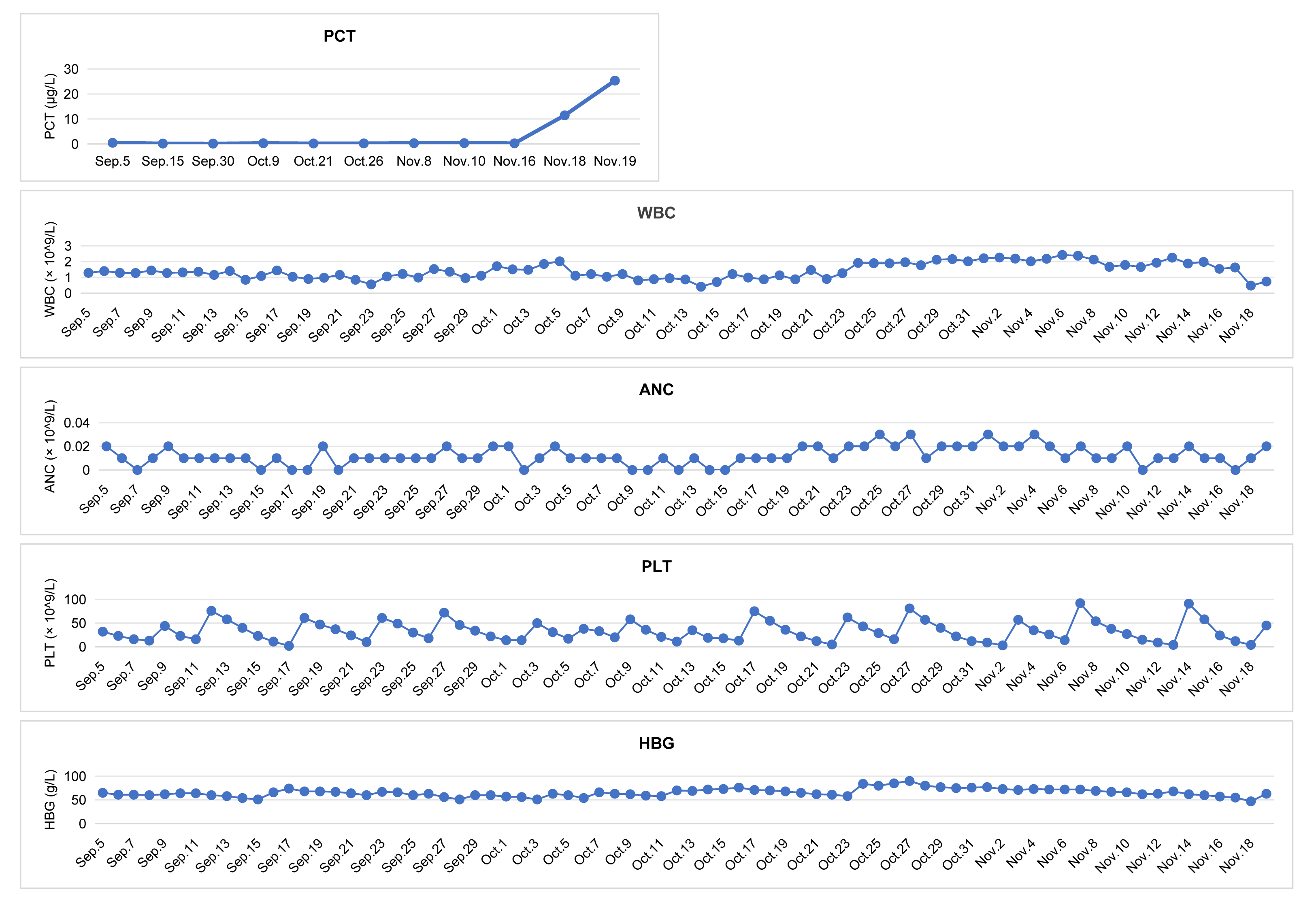


**Supplementary Figure 1**. The results of PCT, WBC, NEUT, PLT, and HBG during hospitalization of the patient. PCT: Procalcitonin; WBC: white blood cells; ANC: absolute neutrophil count; PLT: platelets; HBG: hemoglobin.
